# Impact of reader experience on the time-saving potential of a novel computer-aided tool for Ann Arbor staging of pediatric hodgkin lymphoma

**DOI:** 10.3389/fnume.2026.1843299

**Published:** 2026-06-03

**Authors:** Iryna Vasyliv, Michael Joseph Barrow, Lucia Baratto, Hyun Gi Kim, Vanessa Ricarda von Krüchten, Yashas Ullas Lokesha, Amir Hossein Sarrami, Marine Moeremans, Kip E. Guja, Kristina Elizabeth Hawk, Heike E. Daldrup-Link

**Affiliations:** 1 Department of Radiology, Stanford University, School of Medicine, Stanford, CA, United States; 2 Department of Pediatrics–Hematology/Oncology, Lucile Packard Children's Hospital, Stanford University, Stanford, CA, United States

**Keywords:** 18F-FDG PET/MRI, Ann Arbor staging, computer-aided tool, pediatric hodgkin lymphoma, time efficiency

## Abstract

**Background:**

Accurate staging of Hodgkin lymphoma in children is important for selecting appropriate therapies. However, evaluation of Ann Arbor staging criteria on whole-body ^18^F-FDG PET scans is time-consuming and subject to reader-dependent variability.

**Objective:**

To determine if a novel computer-aided staging tool can improve the time-efficiency and accuracy of Ann Arbor staging.

**Methods:**

^18^F-FDG PET/MRI scans of fifty-six pediatric patients were divided into two groups with matched Ann-Arbor stages. Group 1 was staged via traditional inspection and Group 2 was staged with a newly developed web-based staging tool by one radiology resident, four radiologists and two nuclear medicine physicians. An additional radiologist and nuclear medicine physician jointly established the reference standard. The time to generate the Ann Arbor score was compared between group 1 and 2 with the Wilcoxon Rank-Sum test, and agreement with the reference standard was assessed with Cohen's kappa analysis. In addition, staging time was correlated with reader experience level, measured as the number of PET cases interpreted per year, using linear regression analysis.

**Result:**

Use of the tool significantly reduced Ann Arbor staging time (10.8 ± 6.33 min) compared to traditional staging without the tool (13.87 ± 8.16 min; *p* = 0.001). The greatest reduction in staging time was observed for the radiology resident (17.63 ± 2.3 min with the tool and 21.5 ± 3.3 without the tool), followed by radiologists (13.63 ± 2.5 min with the tool and 18.03 ± 2.6 without the tool), while no significant change was observed for nuclear medicine physicians (1.45 ± 0.82 min with the tool and 1.39 ± 1.12 without the tool). Agreement with the reference standard improved with tool assistance (*κ* = 0.616) compared to traditional staging without the tool (*κ* = 0.945 with the tool). Linear regression demonstrated a significant inverse association between reader experience and time saved using the tool (*β* = −0.812, *p* < 0.001; R² = 0.66), indicating greater benefit for less experienced readers.

**Conclusion:**

The computer-aided staging tool significantly improved time-efficiency and accuracy of Ann Arbor staging, especially for less experienced readers. We provide the tool as a freely accessible web resource.

## Introduction

Hodgkin lymphoma (HL) is among the most commonly diagnosed malignancies in children and adolescents aged 0 to 19 years (1). Timely, accurate diagnosis and staging are essential to guide treatment decisions and achieve optimal outcomes (2). ^18^F-FDG positron emission tomography (PET) is the standard imaging modality for initial staging, treatment response assessment, and surveillance in lymphoma care (3). The Ann Arbor classification, first introduced in 1971, remains the National Comprehensive Cancer Network (NCCN) recommended staging system for HL (4).

However, growing imaging volumes are straining radiology workflows, making thorough and efficient interpretation of complex whole-body ^18^F-FDG PET examinations challenging. Martella et al. reported an increase in ^18^F-FDG PET utilization at major U.S. cancer centers from 5.9 to 6.7 studies per 1,000 persons between 2011 and 2020 (5). Khurana et al. reported that the number of radiologists per 100,000 population remained largely stable, 79.7 in 2012 and 79.9 in 2019 (6), and Morales-Tisnés et al. reported a decline in the number of pediatric radiologists between 2016 and 2023, despite increasing demand for imaging services (7). The widening gap between imaging volume and radiologist capacity is concerning. Klein et al. reported a 20.2 % misdiagnosis rate in on-call pediatric radiology reporting, highlighting a risk to timely and accurate diagnoses (8).

Computer-aided diagnostic (CAD) systems have emerged as a promising response to this bottleneck. Frood et al. demonstrated that AI-assisted lymphoma lesion detection and segmentation integrated into ^18^F-FDG PET/CT staging software reduced reporting time (median 15.0 vs. 13.3 min) without affecting report quality (9). Hasanabadi et al. applied radiomics and machine learning to automatically classify lymphoma on ^18^F-FDG PET scans into early (I–II) vs. advanced (III–IV) stages (10). Thus, current AI approaches assist readers to provide simplified stage grouping. To our knowledge, no existing system fully automates clinical Ann Arbor staging. In addition, previously reported tools were designed as “black box” systems that do not include the reader in the decision-making process (11). In contrast, we designed an interactive tool that guides the reader through the complex staging task, improving efficiency and accuracy without compromising clinician control. The purpose of our study was to determine if this novel computer-aided staging tool can improve the time-efficiency and accuracy of Ann Arbor staging.

## Materials and methods

**Figure 1:**
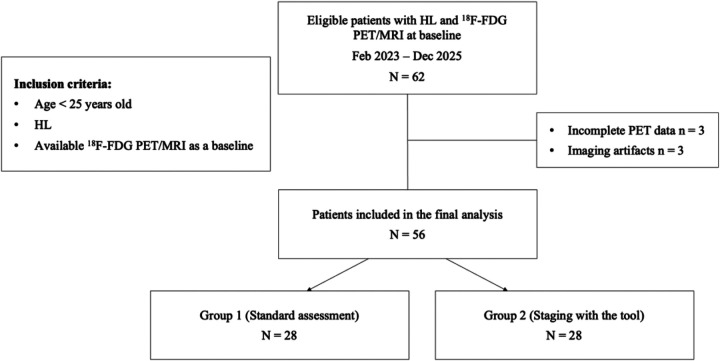
Flow chart depicting the application of inclusion and exclusion criteria, showing the final study population and the allocation of patients to group 1 (standard assessment) and group 2 (staging with AI tool).

This single-center retrospective study was approved by the institutional review board (IRB # 44706) at Stanford University, with a waiver of informed consent due to the retrospective study design. Pediatric and adolescent patients with histologically confirmed Hodgkin lymphoma who underwent baseline ^18^F-fluorodeoxyglucose (FDG) whole-body PET/MRI at Lucile Packard Children's Hospital between February 2023 and December 2025 were identified from an institutional ^18^F-FDG PET/MR image registry. Inclusion criteria were age younger than 25 years, a diagnosis of Hodgkin lymphoma, and availability of baseline ^18^F-FDG PET/MRI performed at initial diagnosis prior to treatment initiation. 62 patients met the eligibility criteria. Six patients were excluded due to incomplete imaging studies (*n* = 3) or severe imaging artifacts (*n* = 3) (Figure 1). The final study cohort comprised 56 patients, including 30 males (53.6%) and 26 females (46.4%), with a mean age of 15.6 ± 3.5 years (range, 7–24 years).

### Image acquisition

All patients underwent whole-body ^18^F-FDG PET/MRI as part of the institutional imaging protocol at Stanford at the time of HL diagnosis, before initiation of cancer therapy. Whole-body ^18^F-FDG PET/MRI is routinely used for pediatric oncology patients to reduce radiation exposure while maintaining high diagnostic accuracy for whole-body staging.

Imaging was performed on a 3-T integrated PET/MRI system (Signa PET/MRI, GE Healthcare, software version MP26) using surface coils tailored to patient size.

Patients fasted for at least 4 h, and blood glucose levels were confirmed to be below 140 mg/dL before tracer administration. ^18^F-FDG was injected intravenously at a dose of 3–5 MBq/kg of body weight. ^18^F-FDG PET/MRI acquisition started approximately 60 min post-injection (mean ± SD, e.g., 58 ± 4 min). The head-to-toe whole-body ^18^F-FDG PET/MRI protocol included the following MRI sequences: Axial T1-weighted fat-saturated LAVA (Liver Acquisition with Volume Acceleration, repetition time TR: 4.2 ms; echo time TE: 1.7 ms; slice thickness SL: 3 mm, no gap). Axial Dixon sequence (water/fat separation) for PET attenuation correction (MRAC, TR: 4.2 ms; TE: 2.2 ms; SL: 5 mm, no gap). Axial Diffusion-Weighted Imaging (DWI, *b*-values: 50 and 800 s/mm², TR: 5,000–8,000 ms; TE: 70–90 ms; SL: 5 mm, interleaved slices). Coronal T2-weighted fast spin echo (thorax and abdomen): TR, 4,500–5,000 ms; TE, 90–100 ms; slice thickness, 4 mm; matrix, 256 × 256; voxel size, 2.8 × 2.8 × 2.8 mm; no gap.

^18^F-FDG PET data were acquired simultaneously with MRI using 3-minute bed positions and an axial field of view of 25 cm. PET images were reconstructed using ordered subsets expectation maximization with time-of-flight and point-spread function modeling (2 iterations, 28 subsets). Attenuation correction of PET data was performed using MR-based attenuation maps derived from a two-point Dixon sequence, which segments tissue into four classes (air, lung, fat, and soft tissue) and assigns corresponding attenuation coefficients for PET reconstruction. Attenuation-corrected PET images were intrinsically coregistered with MRI using GE scanner-integrated software.

### Reference standard

The reference standard for Ann Arbor staging was established by expert consensus. All baseline ^18^F-FDG PET/MRI examinations were independently reviewed by a nuclear medicine physician (L.B.) and a pediatric radiologist (I.V.), both with expertise in oncologic imaging. Readers applied Ann Arbor criteria to the imaging findings to determine the stage, with clinical reports reviewed in parallel to ensure consistency. There were no discrepant interpretations between the expert readers and the clinical reports. This consensus-derived staging assignment served as the reference standard for all subsequent reader evaluations and performance analyses.

### The computer-aided staging tool

**Figure 2:**
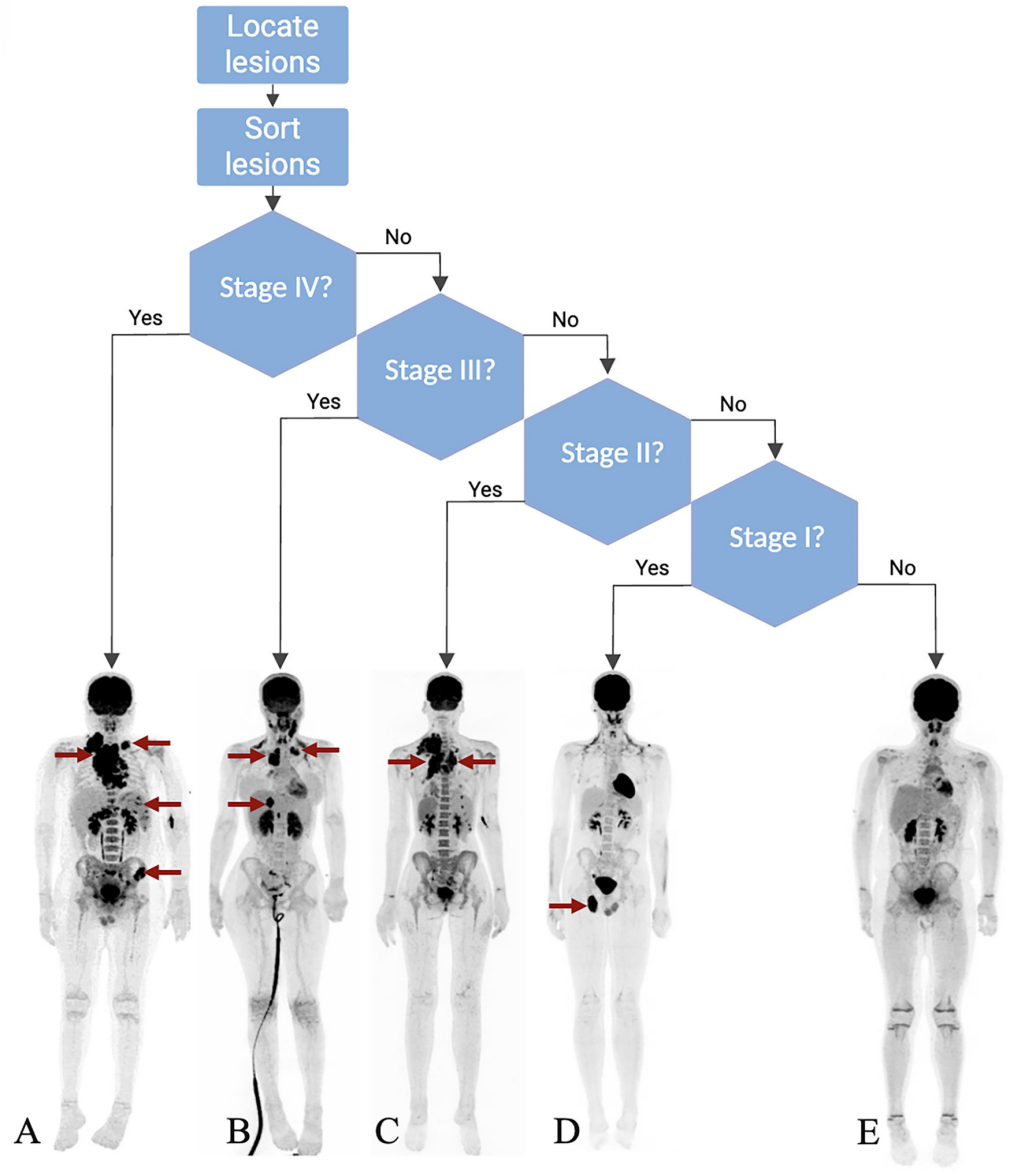
Decision tree for the computer-aided Ann Arbor staging tool. Lesions are first localized and categorized by anatomical distribution, followed by hierarchical evaluation of staging criteria from stage IV to stage I. Representative ^18^F-FDG PET maximum intensity projections (MIP) of four patients with different stages of Hodgin's lymphoma (HL) are shown: **(A)** 11-year-old male with stage IV HL: multiple enlarged lymph nodes above and below the diaphragm, including neck and anterior mediastinum (arrows), with additional hypermetabolic lesions in the spleen (arrow) and left iliac wing (arrow), consistent with splenic and bone marrow involvement. **(B)** 19-year-old female with stage III HL: multiple enlarged lymph nodes above and below the diaphragm (arrows). **(C)** 18-year-old female with stage II HL: multiple enlarged and hypermetabolic lymph nodes above the diaphragm, in the neck and anterior mediastinum (arrows). **(D)** 16-year-old male with stage I HL: single enlarged hypermetabolic lymph node below the diaphragm in the right inguinal region (arrow). **(E)** 10-year-old male with no PET-avid lesions.

A computer-aided staging tool was developed by an experienced computer scientist (M.B.) to support standardized Ann Arbor staging of HL based on PET/MRI findings. The tool combines a rule-based decision tree reflecting Ann Arbor staging criteria with an interactive user interface designed for clinical use (Figure 2). The computer-aided staging tool was implemented primarily in JavaScript with a web-based graphical user interface. Following lesion identification on ^18^F-FDG PET/MRI scans, tumor sites were categorized as nodal or extranodal involvement and assigned to their respective anatomical distribution. A decision tree algorithm then sequentially evaluated staging criteria in descending order, beginning with assessment for stage IV disease, followed by stage III, stage II, and stage I. At each step, a binary decision was made based on imaging-defined tumor extent, such as the distribution of nodal and extranodal tumors relative to the diaphragm. This structured approach enabled deterministic and reproducible staging.

**Figure 3:**
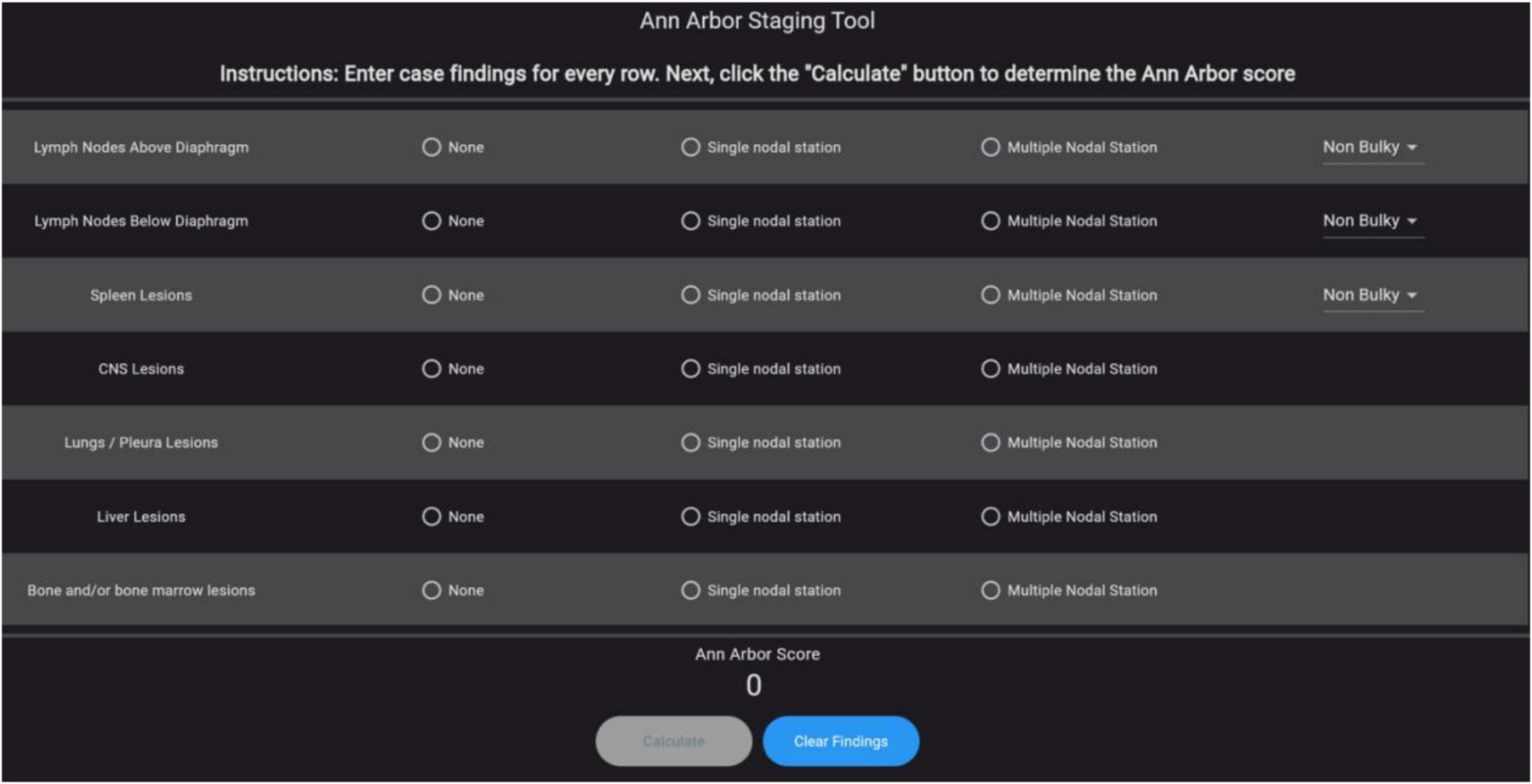
Web-based interface of the Ann Arbor staging tool: the layout for lesion input, anatomical localization, and automated hierarchical staging assessment.

The decision tree was implemented within a web-based graphical user interface designed to reflect routine clinical staging workflows (Figure 3). Users recorded case-specific imaging findings by answering six structured questions corresponding to key anatomical regions: lymph node involvement above and/or below the diaphragm, splenic involvement, and presence of extranodal disease. For each category, disease extent was recorded as none, single nodal station, or multiple nodal stations, with optional designation of bulky disease where applicable.

## Human reader assessments

Fifty-six ^18^F-FDG PET/MRI scans were divided into two groups with matched Ann Arbor stages: Images in Group 1 were staged conventionally, while images in Group 2 were staged with assistance by the web-based computer-aided staging tool. For each case, readers recorded the time to assign an Ann Arbor stage, measured from initial case opening on a PACS workstation to final stage submission.

Seven readers participated, representing a range of training levels and subspecialty backgrounds. These included one second-year radiology resident with no prior PET experience, four board-certified pediatric radiologists with 2, 7, 11 and 15 years of experience, respectively, interpreting 90–1,500 PET examinations per year and two board certified nuclear medicine physicians with 7–9 years of experience, interpreting 2,000–4,500 PET examinations per year.

### Statistical analysis

Staging times without and with the tool were compared using the Wilcoxon rank-sum test. Relative time savings were calculated as the percentage reduction in staging time using the formula: (reading time without tool—reading time with tool)/reading time without tool × 100. For each Ann Arbor stage, staging times were compared using the Wilcoxon signed-rank test. Staging time was correlated with reader experience, measured as the number of PET cases interpreted per year, using linear regression analysis. Agreement between reader-assigned Ann Arbor stage and the reference standard was evaluated using Cohen's kappa coefficient, with interpretation based on the criteria proposed by Landis and Koch (<0, no agreement; 0.01–0.20, slight; 0.21–0.40, fair; 0.41–0.60, moderate; 0.61–0.80, substantial; and 0.81–1.00, almost perfect agreement). Corresponding 95% confidence intervals were calculated for each kappa estimate. All statistical analyses were conducted using SPSS software (version 29.0.2.0; IBM Corp.).

## Results

### Patient demographics

Among the final cohort of 56 patients, the reference standard classified five patients (8.9%) as stage I, 20 patients (35.7%) as stage II, 12 patients (21.4%) as stage III, and 19 patients (33.9%) as stage IV. Bulky disease was present in 12 patients (21.4%), and extranodal involvement was identified in 18 patients (32.1%). Patient characteristics and stage distribution are summarized in Table 1. PET/MRI evaluations for the reference standard identified tumor lesions above the diaphragm in 53 patients (94.6%) and lesions below the diaphragm in 30 (53.6%) patients. Splenic involvement was present in 16 (28.6%) patients, and stage IV with extranodal CNS, lung, liver, bone marrow involvement, or their combination, was noted in 19 patients (33.9%).

**Table 1:** Patient demographics.

Characteristic	Group 1 Patients staged without AI tool	Group 2 Patients staged with AI tool	All patients (*n* = 56)
Age (years)			
Mean ± SD	15.96 ± 3.6	16.21 ± 3.54	15.83 ± 3.53
Range	10–23	7–24	7–24
Sex (number of patients)			
Male	13 (46.4%)	17 (60.7%)	30 (53.57%)
Female	15 (53.6%)	11 (39.3%)	26 (46.43%)
Race (number of patients)			
White	11 (39.3%)	15 (53.6%)	26 (46.4%)
Asian	3 (10.7%)	3 (10.7%)	6 (10.7%)
Black or African American	3 (10.7%)	0	3 (5.4%)
Other	11 (39.3%)	10 (35.7%)	21 (37.5%)
Ethnicity (number of patients)			
Hispanic/Latino	8 (28.6%)	11 (39.3%)	19 (33.9%)
Non-Hispanic/Non-Latino	20 (71.4%)	17 (60.7%)	37 (66.1%)

Patient demographics for Group 1 (scans evaluated without tool assistance) and Group 2 (scans evaluated with the tool assistance). Data are presented as mean ± standard deviation or number (percentage) where appropriate.

### Staging time

**Figure 4:**
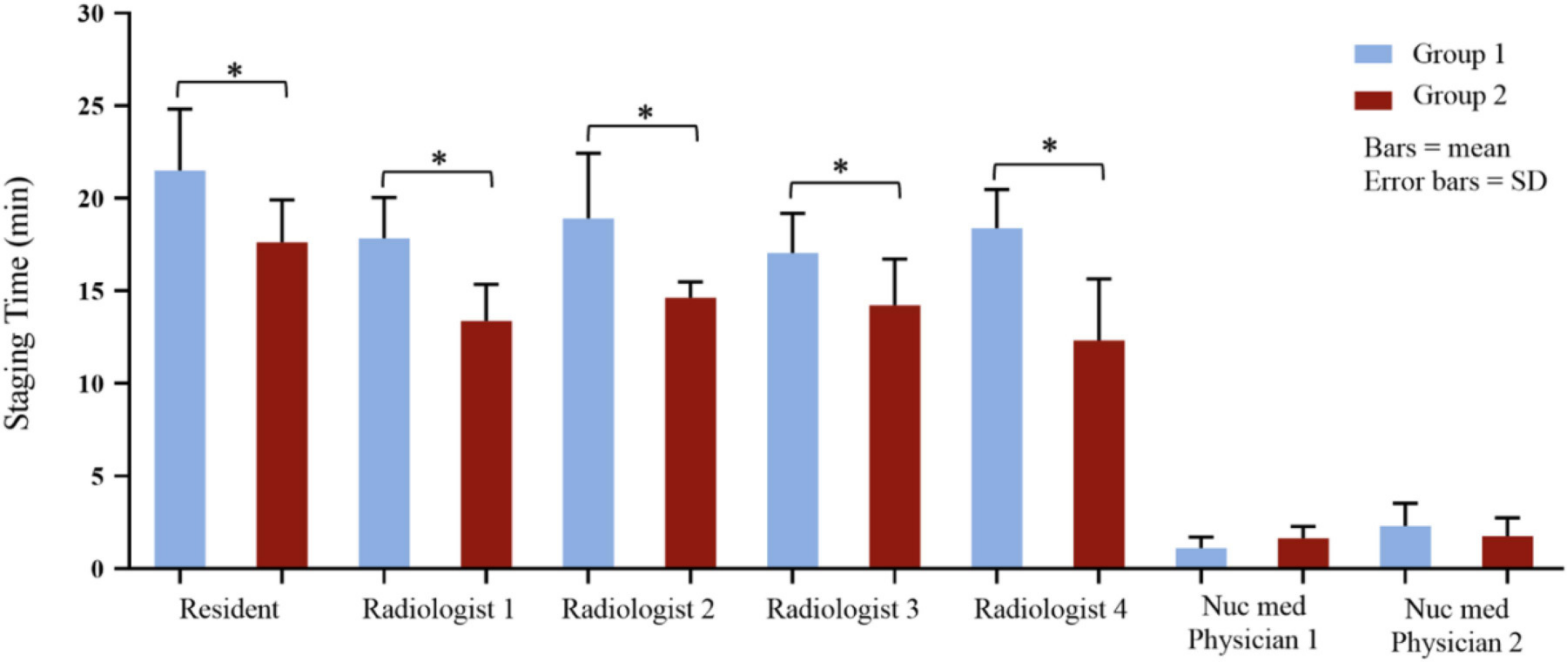
Staging time (minutes) for human readers with and without the computer-aided staging tool: Bar plots show mean staging times for readers with different training backgrounds: one radiology resident, four pediatric radiologists, and two nuclear medicine physicians. Each bar represents the mean staging time across 28 PET/MR examinations per reader. Blue bars represent staging performed without the computer-aided tool, and red bars represent staging with the tool. Error bars indicate standard deviations (SD). Asterisks (*) denote statistically significant differences between staging with and without the tool (*p* < 0.05, paired comparison).

The mean staging time without the tool (Group 1) was 13.87 ± 8.16 min and the mean staging time with the tool (Group 2) was 10.8 ± 6.33 min. The staging times for Group 1 and Group 2 were significantly different (*p* = 0.001). For the second-year resident, the mean staging time decreased significantly from 21.5 ± 3.3 min without the tool to 17.63 ± 2.3 min with the tool (*p* = 0.001). The mean time saved with the tool was 3.88 ± 3.43 min. For the group of four radiologists, the staging time decreased significantly from 18.03 ± 2.6 min without the tool to 13.63 ± 2.5 min with the tool (*p* = 0.001). The mean time saved by using the tool was 4.41 ± 2.32 min. For the two nuclear medicine physicians, the staging time did not change significantly without the tool (1.39 ± 1.12 min) and with the tool (1.45 ± 0.82 min; *p* = 0.554; Figure 4, Table 2).

**Table 2:** Time required for staging patients without tool assistance (group 1) and with tool assistance (group 2).

Reader	Time without the tool (minutes)	Time with the tool (minutes)	*P* value
Resident	21.31 (3.32)	17.27 (2.28)	<0.001
Radiologists			
Reader 1	17.15 (2.15)	13.15 (1.98)	<0.001
Reader 2	17.73 (2.21)	14.57 (0.86)	<0.001
Reader 3	16.50 (2.11)	14.00 (2.49)	<0.001
Reader 4	18.76 (3.51)	12.39 (3.31)	<0.001
Nuc Med			
Reader 1	1.16 (0.58)	1.45 (0.63)	0.554
Reader 2	2.13 (1.22)	1.45 (0.99)	0.154

Data are presented as mean ± standard deviation or as number (percentage). Comparison of mean values between groups was performed using a Wilcoxon Rank-Sum test.

**Figure 5:**
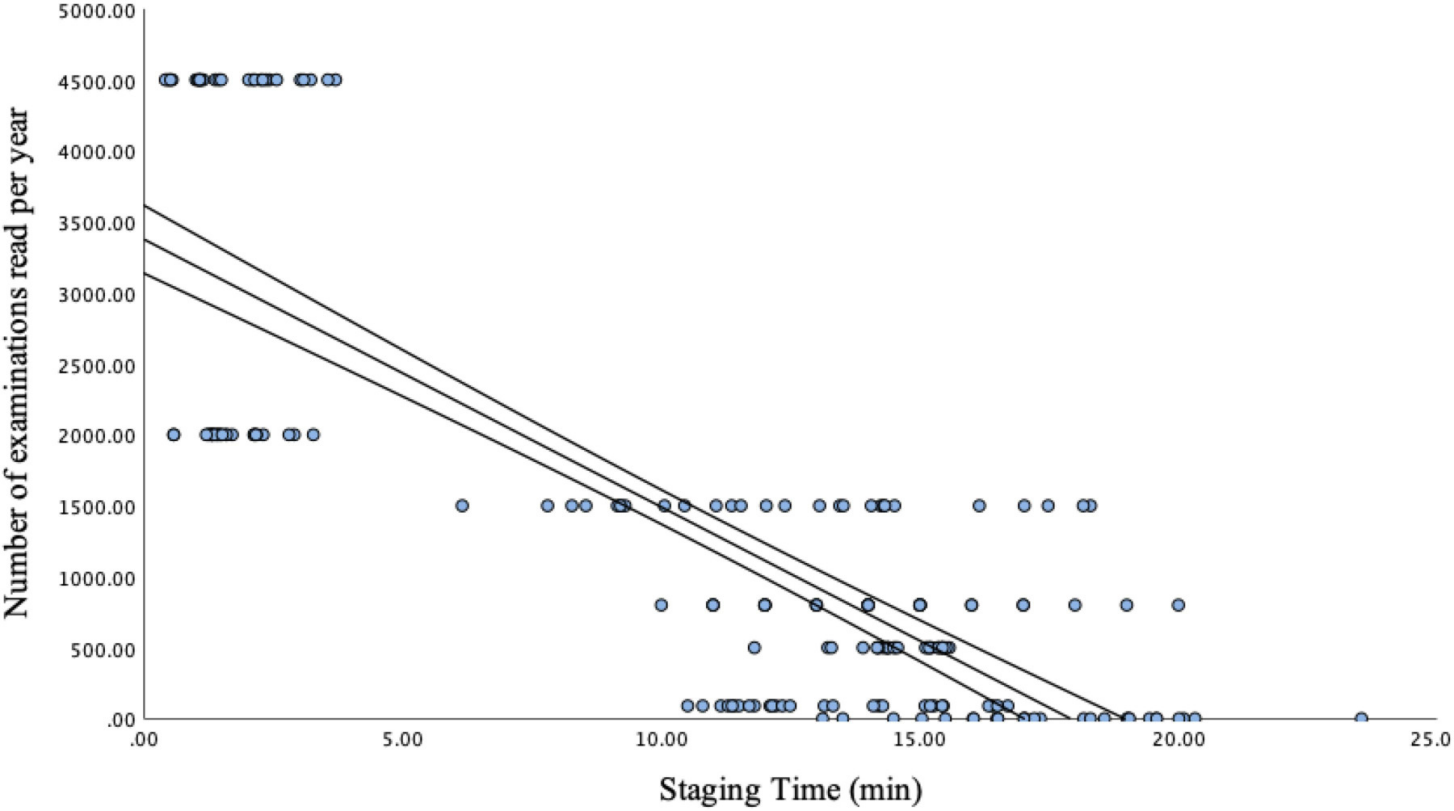
Correlation between staging time using the computer-aided tool and reader experience. Reader experience was measured as the number of PET examinations interpreted per year. The scatter plot shows individual staging times recorded during the evaluation of 28 ^18^F-FDG PET/MR studies by seven readers (blue dots). Higher annual scan volume was significantly associated with shorter staging time using the tool (*β* = −0.812, *p* < 0.001, R² = 0.66). The solid line represents the linear regression with 95% confidence intervals.

Linear regression analysis demonstrated a significant inverse relationship between reader experience, measured as annual PET interpretation volume, and the time saved by using the tool, measured as the difference in staging time with and without the tool (*β* = −0.812, *p* < 0.001, R² = 0.66; Figure 5).

#### Relative time savings across Ann Arbor stages

Comparison of mean staging times across Ann Arbor stages demonstrated stage-dependent time savings with the tool. The largest relative reductions in staging time were observed for stage I and stage IV disease, with reductions of 36.13% and 36.30%, respectively. For stage I disease, the mean staging time decreased significantly from 16.9 ± 9.14 min without the tool (Group 1) to 12.42 ± 4.52 min with the tool (Group 2; *p* = 0.021). For stage II disease, staging time decreased by 22.04%, from 16.0 ± 6.12 min without the tool to 13.1 ± 4.77 min with the tool (*p* = 0.024). For stage III disease staging time decreased by 23.42%, from 17.68 ± 9.58 min without the tool to 14.33 ± 7.01 with the tool (*p* = 0.028). For stage IV disease, the mean staging time decreased from 20.67 ± 7.35 min without the tool (Group 1) to 15.17 ± 5.29 min with the tool (Group 2, *p* = 0.033).

### Agreement with the reference standard

Agreement between reader-assigned Ann Arbor stage and the reference standard for each reader is summarized in Table 3. Without tool assistance, agreement varied by reader expertise, with lower agreement among less experienced readers and consistently high agreement among expert readers.

**Table 3:** Interobserver agreement with the reference standard for readers performing staging with and without the computer-aided tool.

Reader	Kappa without the Tool	S.E	Confidence interval	Kappa with the Tool	S.E	Confidence interval
Resident	0.433	0.12	0.184–0.681	0.789	0.10	0.586–0.933
Radiologist 1	0.476	0.12	0.230–0.721	0.946	0.05	0.836–1.000
Radiologist 2	0.524	0.12	0.279–0.769	0.789	0.10	0.586–0.933
Radiologist 3	0.525	0.11	0.297–0.752	0.620	0.13	0.363–0.878
Radiologist 4	0.588	0.12	0.343–0.833	0.643	0.11	0.412–0.873
Nuclear med physician 1	0.836	0.09	0.655–1.000	0.946	0.05	0.836–1.000
Nuclear med physician 2	0.834	0.09	0.654–1.000	0.947	0.05	0.838–1.000

Agreement is expressed as Cohen's kappa, with standard error (S.E.) and 95% confidence intervals (CIs).

The radiology resident demonstrated the lowest baseline agreement and the highest number of staging discrepancies, with 11 errors without the tool (*κ* = 0.433) and 4 errors with tool assistance (*κ* = 0.789). Among radiologists, Radiologist 1 demonstrated the largest improvement among radiologists, with discrepancies decreasing from 10 errors (*κ* = 0.476) to 1 error (*κ* = 0.946). Radiologist 2 improved from 9 errors (*κ* = 0.524) to 4 errors (*κ* = 0.789), and Radiologist 3 showed a reduction in discrepancies from 9 errors without the tool (*κ* = 0.525) to 7 errors with the tool (*κ* = 0.620). Radiologist 4 showed a smaller change, with 8 errors without the tool (*κ* = 0.588) and 7 errors with the tool (*κ* = 0.643). Nuclear medicine physicians demonstrated high baseline agreement with the reference standard, with 3 errors each without the tool (*κ* = 0.836 and 0.834), which decreased to 1 error each with tool assistance (*κ* = 0.946 and 0.947). Overall, the staging tool reduced the number of discrepancies with the reference standard for the resident and less experienced radiologists, but not expert readers. These findings indicate that the staging tool primarily benefits less experienced readers, while providing limited added value for expert readers who already perform at a high level.

## Discussion

Our results showed that a novel computer-aided staging tool can improve the time-efficiency and accuracy of Ann Arbor staging, particularly for readers operating outside highly optimized expert workflows. Ann Arbor staging plays a key role in risk stratification and treatment planning in pediatric Hodgkin lymphoma. However, inconsistencies in staging results have been noted between different readers. Singh et al. reported discordance rates of 11.3% in disease staging and 7.8% in risk stratification between a multidisciplinary clinic and an independent expert team. In all discordant cases, there was up-staging of patients by the multidisciplinary clinic which led to overtreatment of the patient (16). Zijlstra et al. reported only 56%–61% agreement between Ann Arbor staging of baseline PET examinations by eleven nuclear medicine physicians with varying experience and expert readers (17). These findings highlight the dependence of staging accuracy on reader experience, underscoring the need for computer-aided decision-support tools to standardize Ann Arbor staging and reduce interobserver variability.

Supporting this concept, a recent multicenter study by Jemma et al. demonstrated that a fully automated deep learning algorithm for Lugano response assessment in FDG-avid lymphomas achieved agreement rates of 93%, 87%, and 85% with adjudicated expert radiologic responses across three independent clinical trial cohorts (18), suggesting that automated approaches may help reduce reader-dependent variability in lymphoma imaging assessment (18). Similarly, Sadik et al. developed an AI-based tool for automated detection of bone marrow ^18^F-FDG uptake in HL patients, achieving 81% agreement with expert readers (19). These findings demonstrate that automated and computer-aided approaches can achieve expert-level performance in complex lymphoma imaging tasks.

A systematic literature review of explainable artificial intelligence in medical image analysis by Muhammad and Bendechache comprehensively evaluated existing Explainable Artificial Intelligence approaches and highlighted that the “black-box” nature of deep learning models remains a major barrier to clinical adoption, particularly because model decisions lack transparency and interpretability in high-stakes medical settings (12). Importantly, this lack of transparency is not merely a theoretical limitation but may have direct clinical consequences. Prior studies have demonstrated that deep learning models can rely on non-causal or confounding features, thereby limiting generalizability and potentially affecting clinical reliability. For example, Zech et al. showed that a deep learning model for pneumonia detection achieved significantly reduced performance when applied to external datasets, as it had inadvertently learned to depend on imaging device characteristics rather than disease-specific features (13). Similarly, DeGrave et al. demonstrated that AI models for COVID-19 detection may rely on spurious correlations unrelated to pathology, further highlighting the risk of shortcut learning (14).

Supporting this, research by Singla et al. introduced a counterfactual explanation framework for medical image classification and demonstrated that conventional explanation methods, such as saliency maps, are insufficient because they only indicate where the model focuses but do not explain what imaging features drive the prediction or how changes in these features affect the decision (15). Importantly, in a human-grounded evaluation with radiology trainees, counterfactual explanations significantly improved users' understanding of model decisions compared with standard approaches (15).

In contrast, the proposed computer-aided staging tool follows a transparent, rule-based (“white-box”) approach, in which each decision step is explicitly defined and guided by established clinical criteria. This allows direct verification of the decision-making process and ensures that staging outcomes are derived from clinically relevant inputs. By integrating domain knowledge into the decision pathway, the proposed system improves interpretability, reproducibility, and ultimately the clinical reliability of imaging-based staging.

Peters et al. performed a comparative analysis of two CAD systems and demonstrated substantial variability in volumetric measurements, with relative volume differences of 1.3 ± 18.6% for solid nodules and −15.2 ± 23.5% for ground-glass nodules, resulting in discordant lesion classification in 14.9% of cases and significant differences in correct classification rates (88.5% vs. 79.8%, *p* = 0.004) (20). These findings highlight that variability in CAD-derived measurements can directly affect reproducibility and, consequently, the clinical reliability of imaging-based decision-making. In this context, our tool not only improves efficiency but also supports a structured and standardized staging workflow, which may reduce variability in interpretation and improve consistency across readers.

Wu et al. evaluated the impact of CAD on inter-observer agreement in nodule management and demonstrated a significant improvement in reproducibility, with Fleiss *κ* increasing from 0.437 to 0.623 (21). The use of CAD reduced overall management discrepancies from 39.0% to 26.2%, including a reduction in major discrepancies from 27.5% to 15.8% and substantial discrepancies from 4.8% to 1.5% (21). In addition, CAD improved detection sensitivity in part-solid nodules from 82.6% to 92.2% (*p* < 0.05), supporting its role in enhancing both clinical reliability and diagnostic performance.

Several computer-aided diagnosis approaches have been proposed to address specific components of lymphoma imaging interpretation. Yang et al. developed deep learning–based ^18^F-FDG PET/CT models to differentiate lymphomatous involvement from metastatic disease in enlarged cervical lymph nodes, achieving diagnostic accuracies of up to 86.9%, thereby supporting more reliable nodal classification during staging (22). Gao et al. introduced a longitudinal image navigation and analysis system for automated lesion SUV measurements on serial PET/CT scans of lymphoma patients and demonstrated close agreement with radiologist-derived measurements, with an average relative squared difference in SUVmax of 0.02 (23). Yu et al. proposed a semi-automated PET/CT workflow in which CT-based multi-atlas segmentation is first used to remove organs with physiologic tracer uptake, followed by conditional random field-based detection and segmentation of candidate lymphoma volumes on PET images, with final user selection of true-positive lesions. In their cohort of 11 patients, the approach demonstrated 100% detection of all reference lymphoma regions and achieved a mean Dice coefficient of 84.4% compared with manual segmentations, supporting automated tumor volume delineation for assessment of disease burden (24). Complementing lesion detection and quantification approaches, Sollini et al. proposed a radiomics framework demonstrating that analysis incorporating all lesions, rather than a single target lesion, improved classification of relapsing/refractory vs. non-relapsing disease, achieving an accuracy of 82%, highlighting the importance of whole-disease characterization for outcome stratification beyond conventional staging (25). Together, these studies illustrate how computer-assisted tools can support nodal classification, quantitative disease assessment, lesion segmentation, and whole-disease characterization, thereby addressing multiple steps of lymphoma staging and response evaluation.

Beyond diagnostic performance, the integration of AI tools into clinical workflows has been explored with respect to efficiency and reader experience. Müller et al. reported that a PACS-integrated AI tool for chest CT interpretation did not significantly increase reading time for either residents or consultants (resident: 370 s without AI vs. 437 s with AI; consultant: 366 s vs. 380 s), while providing additional actionable findings in 12.5% of studies (26). Brown et al. used a commercial chest CT CAD system to automate lesion measurements and reporting in clinical practice, reducing reporting time by 7%–44% compared with manual methods (27). Frood et al. demonstrated that AI-assisted PET/CT reporting achieved diagnostic performance comparable to standard interpretation while modestly reducing reporting time from a median of 15.0 to 13.3 min (9). Similarly, Xia et al. reported that a CT-based clinico-radiomics human-machine hybrid system improved diagnostic performance in distinguishing mediastinal lymphoma from thymic epithelial tumors by 14%–40%, with the greatest gains observed among junior radiologists (28).

Our study focused on lymphoma staging, while most prior CAD systems focused on lesion detection, segmentation, or quantitative assessment. For example, Lartizien et al. developed a computer-aided diagnosis system for automated lesion detection on ^18^F-FDG PET/CT of adult patients with lymphoma (29). Using a combination of PET and CT first order and textural features, their machine-learning classifiers achieved high diagnostic performance, with AUC values up to 0.97 for distinguishing malignant from physiologic and inflammatory regions. While demonstrating the feasibility of CAD-based lesion characterization in lymphoma, this approach did not address Ann Arbor staging (29). Wang et al. developed one of the first AI-based approaches for lymphoma detection in pediatric patients on combined ^18^F-FDG PET and T1-weighted MRI scans. Lesions suspicious for tumor were initially identified on PET based on areas of increased FDG uptake. False-positive regions were subsequently excluded using a CNN-based classifier that evaluated both metabolic PET signal and anatomical MRI appearance to distinguish true tumor lesions from physiologic uptake and normal structures (30). Hasanabadi et al. developed a radiomics-based machine-learning framework for automated Lugano staging in adults using PET/CT, achieving an AUC of 0.87 and a sensitivity of 0.88 in external validation (10). Our staging tool was tested on a cohort of pediatric patients. In the future, it could also be applied to adult patients. The tool provides a structured interface that allows clinicians to enter imaging findings and it automatically assigns an Ann Arbor stage in a standardized manner. This approach supports consistent staging by guiding clinicians through structured input of nodal and extranodal involvement, ensuring systematic consideration of nodal regions, organ involvement, and bulky disease, thereby reducing variability in staging decisions and facilitating consistent reporting across readers with different levels of experience. Importantly, our proposed CAD system differs from previously reported approaches by directly addressing the clinical staging workflow rather than isolated tasks such as lesion detection or segmentation. While prior tools primarily assist in identifying or quantifying disease, our system is designed to guide the clinician through the full Ann Arbor staging process using a structured and interactive framework. This allows standardized integration of imaging findings into a clinically meaningful staging output, representing a shift from automated prediction toward clinician-guided decision support. From a clinical perspective, improved standardization of Ann Arbor staging may have direct implications for patient management. Accurate staging determines treatment intensity, including the choice between limited-field therapy and more aggressive combined-modality regimens. Reducing interobserver variability may therefore decrease the risk of both over- and undertreatment, which has been previously reported in discordant staging scenarios. In addition, a structured and guided staging workflow may be particularly valuable in settings with limited subspecialty expertise, supporting more consistent decision-making across institutions. By integrating imaging findings into a standardized staging output, the proposed tool has the potential to improve the reliability of risk stratification and facilitate more uniform treatment planning in pediatric Hodgkin lymphoma.

## Conclusion

The novel computer-aided staging tool presented here can improve the time-efficiency and accuracy of Ann Arbor staging, particularly for readers with limited experience. By reducing variability and accelerating report generation, it improves patient care and decreases workload for radiologists. To promote broad accessibility and widespread use, we are offering this tool as a freely accessible web resource to clinicians and researchers worldwide: https://mike-barrow.github.io/wbtdemo/.

## Data Availability

The raw data supporting the conclusions of this article will be made available by the authors, without undue reservation.
